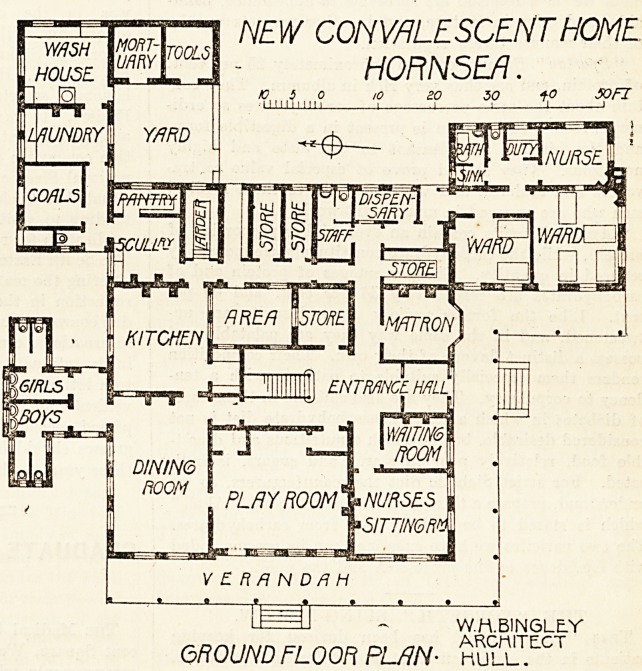# Convalescent, Home, Hornsea

**Published:** 1908-12-12

**Authors:** 


### HOSPITAL ADMINISTRATION.

### CONSTRUCTION AND ECONOMICS.

### CONVALESCENT HOME, HORNSEA.

This Home, which has recently been erected, is a branch of the Victoria Hospital for Sick Children, Hull; and is built on the north cliff, facing the sea. It is over twenty years since the first Convalescent Home was opened with accommodation for eight or nine patients. This was replaced in 1904 by a larger house in which fourteen children could be housed; and now a third Home has supplanted the former ones with a total accommodation for thirty patients.

The building is compactly planned, with the patients' rooms mainly facing south or west. On the ground floor on the south and west sides a verandah with a glass roof is provided as an exercise-ground in bad weather. Besides the usual domestic and administration offices there are on this floor a large play-room for the children, with the dining-room adjoining. Approached from the dining-room and also from the garden are lavatories and w.c.'s for boys and girls respectively. An open lobby effectually cuts off these offices from the interior of the building.

At the south-east angle of the main building, in a one-story wing, are two small wards, one for four, the other for two, beds, with nurses' room, small duty-room, bath-room, w.c., and sink-room. These rooms are intended for special cases and if necessary for isolation; and the only access from the main building is by a lobby, one side of which is open to the air. The only blot on an otherwise excellent arrangement is the position of the w.c., which has no sort of cut-off lobby. A one-story wing on the north-east provides a small laundry and a mortuary.

The upper floor contains the dormitories for children and the bedrooms for staff. Judging by the sizes of the two dormitories for patients it would appear that the boys considerably outnumber the girls. The bath-room and w.c. for boys are cut off from the corridor by a cross-ventilated lobby, while the two w.c.'s for girls are not so arranged; neither are those for the staff. In an otherwise excellent plan these mistakes are much to be regretted. It should be a rule in all buildings of this kind that all w.c.'s should be put in projecting towers with cut-off lobbies. On the other hand the bath-rooms should be as close as possible to the dormitories.

The building was designed and carried out under the superintendence of Mr. W. H. Bingley, who was the architect of the present institution.

## Figures and Tables

**Figure f1:**
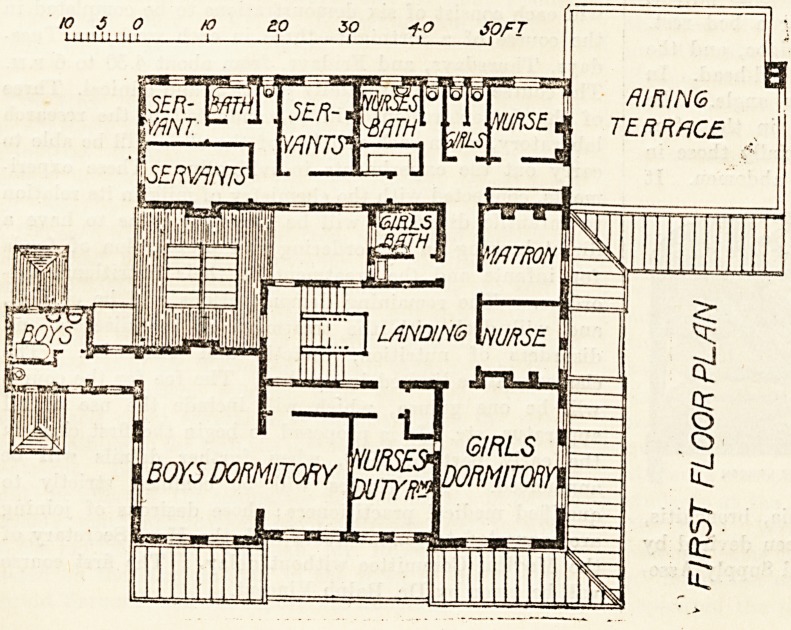


**Figure f2:**